# Performance Analysis of Ferronickel Slag-Ordinary Portland Cement Pervious Concrete

**DOI:** 10.3390/ma17071628

**Published:** 2024-04-02

**Authors:** Zhongping Tang, Hua Peng, Pingbo Mei, Fanglin Huang, Shixiang Yi, Fan Feng

**Affiliations:** 1 Institute of Structural Material Failure and Strengthening Technology, Ningbo Polytechnic, 388 East Lushan Rd., Ningbo 315800, China; 17311158886@163.com; 2 National Engineering Laboratory for Applied Technology of Forestry & Ecology in South China, Central South University of Forestry and Technology, 498 South Shaoshan Rd., Changsha 410075, China; 20220100066@csuft.edu.cn; 3 Zhoushan National Ocean Fisheries Base Infrastructure Construction and Development Co., Ltd., 1 Shanghui Rd., Zhoushan 316291, China; 4 School of Civil Engineering, Central South University, 22 South Shaoshan Rd., Changsha 410075, China; 5 School of Architectural Engineering, Hunan Institute of Engineering, Xiangtan 411100, China; ff-fengfan@163.com

**Keywords:** Ferronickel slag, pervious concrete, compressive strength, permeability coefficient, porosity, composited materials

## Abstract

It is unknown whether Ferronickel slag (FNS)-ordinary Portland cement (OPC)-based pervious concrete (FOPC) is feasible. To this end, a feasibility study was conducted on FOPC. Firstly, a detailed microscopic examination of the FNS powder was conducted, encompassing analyses of its particle size distribution, SEM, EDS, and chemical composition. These analyses aimed to establish the suitability of a composite of FNS and OPC as a composite cementitious material. Subsequent experimentation focused on evaluating the compressive strength of the composite paste material with varying mixed proportions, revealing a slight reduction in strength as the FNS substitution rate increased. Furthermore, the study designed eighteen different mix proportions of FOPC to investigate the key physical properties, including porosity, density, compressive strength, and the coefficient of permeability. Findings indicated that increases in the cementitious material proportion correlate with enhanced concrete strength, where the ratio of cementitious to aggregate increased by 6.7% and 16.5%, and the strength of FOPC increased by 10–13% and 30–50%, respectively. Conversely, a rise in the FNS substitution rate led to a reduction in compressive strength across different mix ratios. Additionally, the ratio of paste material to aggregate was found to significantly influence the permeability coefficient. These comprehensive performance evaluations suggest that incorporating FNS into OPC for pervious concrete applications is a feasible approach, offering valuable insights for the promotion of waste reuse and the advancement of energy conservation and emissions reduction efforts.

## 1. Introduction

As urban development progresses, road waterlogging emerges as a significant municipal challenge. Concurrently, the proliferation of concrete pavements is substantially affecting urban underground ecosystems. To mitigate these issues, the adoption of pervious concrete pavement is gaining advocacy.

A variety of studies have been conducted to propose novel techniques for improving pervious concrete materials [1,2,3,4]. Research by Zheng et al. [5] focused on the creation of alkali-activated metakaolin pervious concrete, incorporating copper slag as an innovative component. Wu et al. [6] investigated the impact of aggregate morphology on the mechanical strength and permeability of pervious concrete. Teymouri et al. [7] examined the properties of adsorbent pervious concrete for potential use in the treatment of refinery and wastewater, aiming to enhance its application as a tertiary treatment method. Research by Tang et al. [8] resulted in the preparation of carbonated recycled aggregate pervious concrete through two distinct carbonation methods. In the investigation conducted by Nazeer et al. [9], the substitution of standard Portland cement with materials such as fly ash and Silica Fume was scrutinized for its effects on the structural integrity, endurance, and microscopic characteristics of pervious concrete. The effects of substituting a portion of ordinary Portland cement (OPC) with diatomaceous earth for porous concrete applications were investigated by Alex et al. [10], with a focus on assessing its impact on various strength parameters. Gao et al. [11] investigated the practicality of incorporating ceramsite into pervious concrete mixtures, highlighting its advantages for constructing infrastructure that is both more eco-friendly and robust. Mitrosz et al. [12] conducted a review on the innovative use of recycled concrete aggregates and rubber particles from waste tires as sustainable alternatives to traditional natural aggregates in part. Park et al. [13] studied the characteristics of strength and permeability in pervious concrete that incorporated coal bottom ash aggregates, creating mixes with diverse aggregate size distributions. Anwar et al. [14] analyzed the performance of pervious concrete with various substitutes for cement and aggregates, and the results showed that using substitutes is feasible. Martins Filho et al. [15] proposed a dimensionless permeability parameter that can fully and clearly classify the flow resistance provided by each sample.

Ferronickel slag (FNS), a granular solid waste, is produced during the industrial process of extracting nickel and iron from their ores. The primary sources of nickel–iron include laterite nickel ore (oxidized nickel ore) and sulfide nickel ore, with the former being more abundant and easier to mine and transport, hence its widespread use and development. As the scale of Ferronickel smelting expands and the production of metallic nickel increases in Chine, the generation of waste FNS has also escalated. Therefore, the resourceful and effective utilization of FNS is imperative, holding significant importance for resource conservation, environmental protection, and sustainable development.

The primary chemical composition of FNS closely resembles that of the OPC. Consequently, numerous scholars have suggested incorporating FNS as a mineral admixture in concrete. For example, research by Sun et al. [16] delved into the impact of replacing traditional sand with FNS in concrete, while keeping the superplasticizer quantity unchanged. Bao and his team [17] investigated the compressive strength and transport mechanisms of concrete that use recycled aggregates with FNS as a fine component, revealing that blends with 40% to 50% FNS showcased enhanced compressive strength and improved resistance to transport phenomena. Kim and colleagues [18] experimented with the use of FNS powder as an alternative to ordinary Portland cement, testing three different FNS variants for their efficiency as binders. More research on FNS-based concrete or FNS-based cementitious material can be found in the literature [19,20,21,22].

The research findings discussed above suggest that FNS can partially substitute cement as a cementitious material. Moreover, FNS ordinary concrete and its building components have also been applied and studied. However, the amount of concrete used in municipal roads is also large, including highways, bridges, pedestrian walkways, etc., which may require the use of pervious concrete. However, due to the significant difference in mix proportion between pervious concrete and ordinary concrete, i.e., the absence of fine aggregates in pervious concrete and the need to meet both strength and permeability performance, it is unknown whether FNS-OPC pervious concrete (FOPC) can be applied. Utilizing FNS in the production of FOPC bears significant implications for managing industrial FNS smelting waste and reducing carbon emissions. Consequently, the objective of this paper is to explore the development of FOPC and to experimentally evaluate its fundamental mechanical and permeability characteristics.

## 2. Materials and Testing

### 2.1. Raw Materials

**Figure 1:**
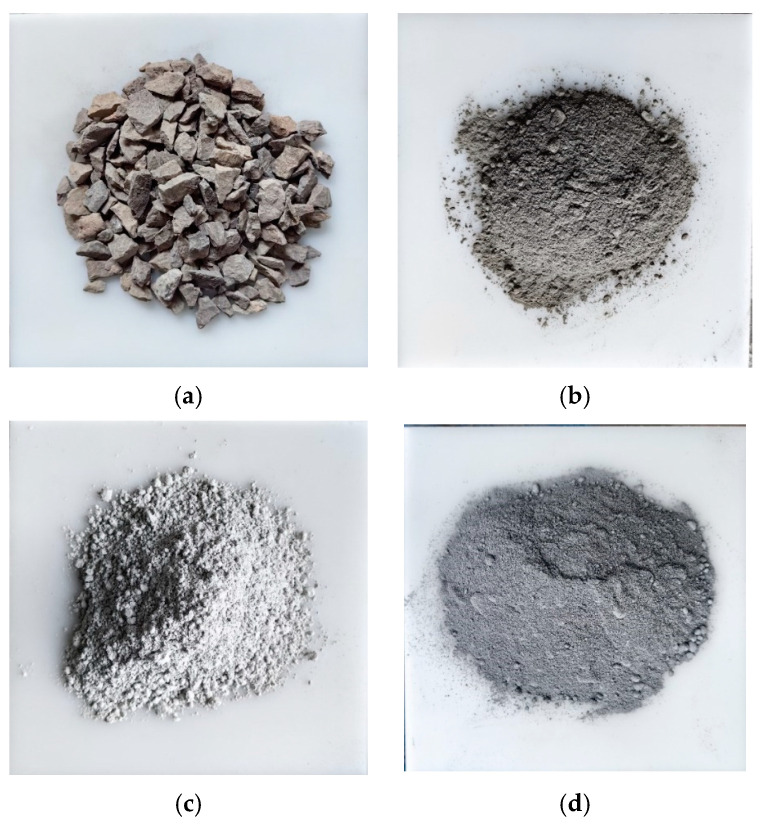
Raw materials: (**a**) coarse aggregate; (**b**) OPC; (**c**) reinforcing agent; and (**d**) FNS.

Basalt stones, with a particle size of 10–20 mm, were used as aggregates for pervious concrete, meeting the requirements of standard JTGE42-2005 [23], as shown in Figure 1a. The cement used was OPC, as depicted in Figure 1b. In order to improve the bonding performance between paste materials and aggregates, interface-reinforcing agents were added to concrete, as shown in Figure 1c. This interface-reinforcing agent was produced by Nanjing Hainiu Company, Nanjing, China, and was a special product for preparing pervious concrete, which is rich in nano silica (SiO_2_) active particles and multi element high functional materials. It was processed through multiple processes such as grinding, drying, and mixing. The FNS powders, originating from Fuzhou, China, are shown in Figure 1d as a gray powder.

**Figure 2:**
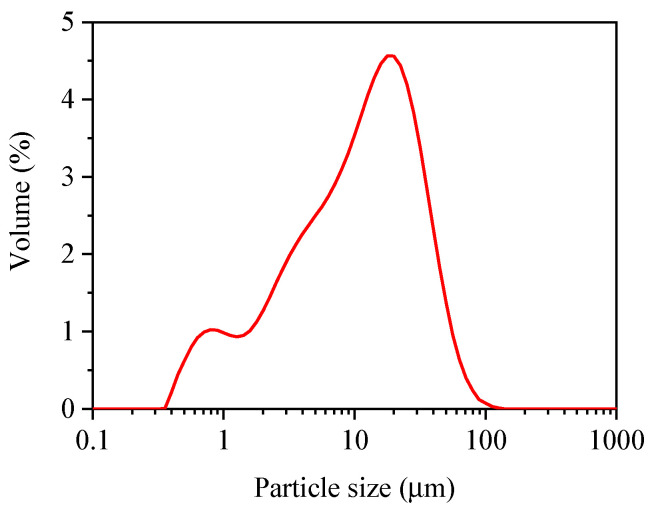
Particle size distribution of FNS.

**Figure 3:**
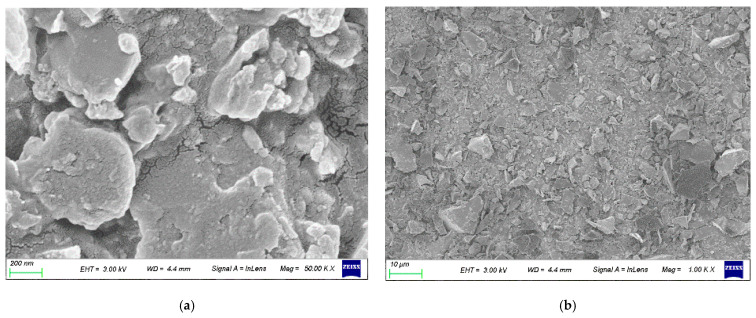
SEM of the FNS: (**a**) mag. 50,000×; (**b**) mag. 1000×.

**Figure 4:**
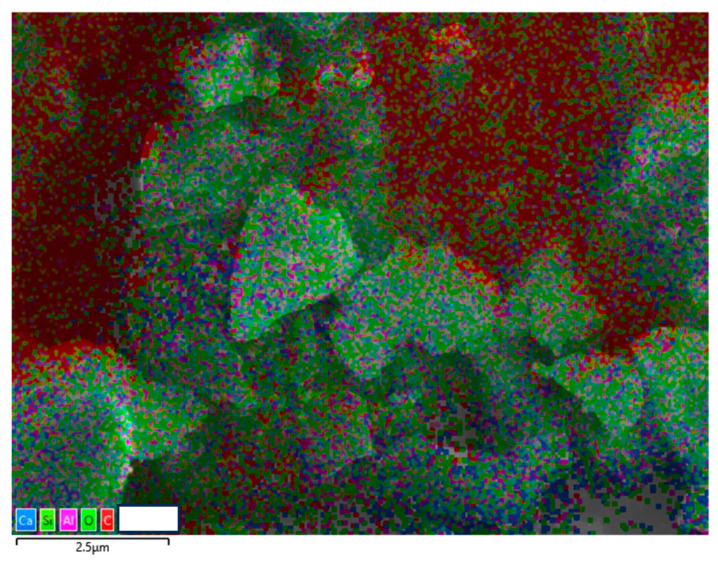
EDS at mag. 5000× of the FNS.

The particle size distribution was tested by a Laser Particle Size Analyzer called Mastersizer 3000, London, UK, and this instrument uses laser diffraction technology to measure particle size. Figure 2 presents the particle size distribution of FNS powder, ranging from 0.5 to 100 μm, similar to Metakaolin [5]. A scanning electron microscope machine called Phenom Pro was used to observe the microscopic structure of FNS, and the images of FNS are displayed in Figure 3, and it contains FNS images at different magnification (Mag). The EDS Mag 5000× of FNS is shown in Figure 4.

A X-ray fluorescence analyzer (XRF) was used to test the detail component of FNS, and the main steps are as follows: firstly, the powder sample was subjected to drying treatment; after drying, the sample was crushed and ground; then, a plastic ring was used to fill the powder, and the compressed sample was directly pressed on a hydraulic press; lastly, the compressed sample was placed in a sample box and tested in a vacuum environment. The results are shown in Table 1 and indicate that the main constituents of FNS are CaO, SiO_2_, Al_2_O_3_, and MgO. These components, comparable to other mineral admixtures, undergo hydration reactions and can be alkali-activated by the alkaline substances in OPC. This underpins the formation of FNS-OPC composite cementitious materials [24,25,26,27,28].

### 2.2. Mix Proportions

To examine the mechanical properties and permeability of FOPC and evaluate the effects of diverse mixing ratios and FNS amounts on its functionality, the properties of concrete with different mix ratios were studied, and the precise mix designs are delineated in Table 2; the designs mainly accounted for the balance between cementitious substances and aggregates, as well as the proportion of FNS used.

Three groups of FOPC were designed, and the difference between each group is the difference in the ratio of cementitious materials to aggregates, including 0.1875, 0.2000, and 0.2188. The OPC and FNS constituted the cementitious base, employing a methodology for replacing OPC with FNS, and each group contains six kinds of FSN substitution rates, including 0, 10%, 20%, 30%, 40%, and 50%; in total, eighteen mix designs for FOPC were considered.

In order to test the strength of the FNS-OPC-based paste material (FOPM), the FOPM test blocks were prepared while the concrete test blocks were made, and its mix proportions are shown in Table 3, where the mix proportions corresponded to the paste material used in concrete.

### 2.3. Preparation of Test Blocks

**Figure 5:**
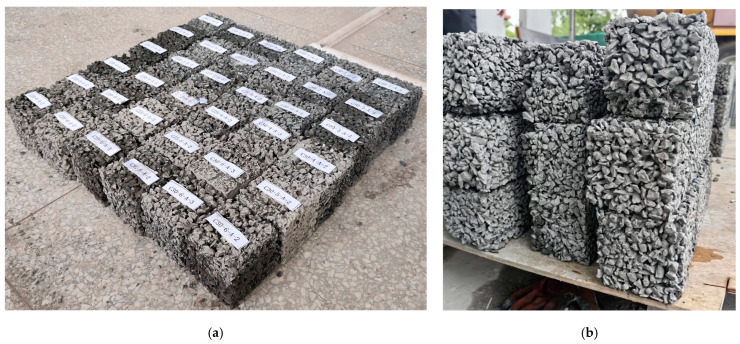
Parts of the FOPC test blocks: (**a**) overall photo; (**b**) detailed photo.

**Figure 6:**
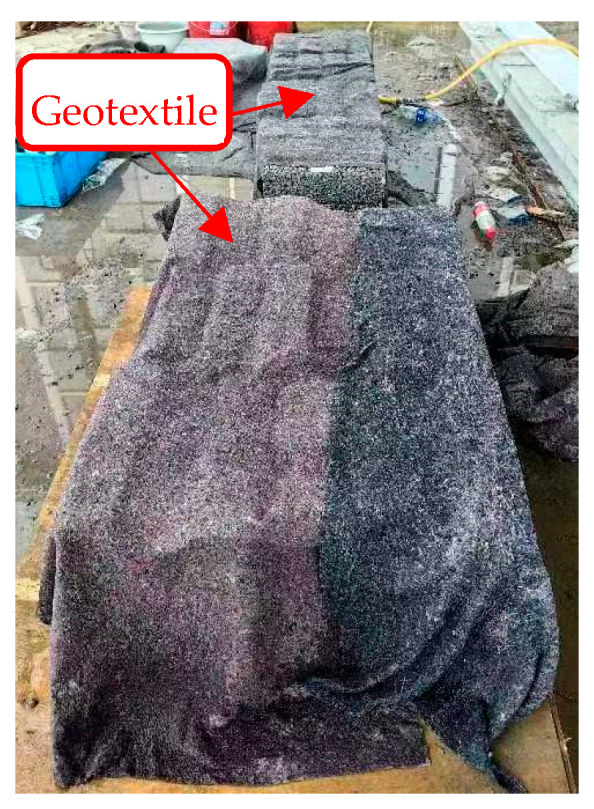
Curing of test blocks.

For evaluating the mechanical properties of the FOPM, cubic test blocks with dimensions of 70.7 mm × 70.7 mm × 70.7 mm were fabricated according to the guideline JGJ/T70-2009 [29], and sixteen test blocks were prepared for each mix proportion. Similarly, cubic test blocks of FOPC, measuring 100 mm × 100 mm × 100 mm, were constructed to assess the basic properties, including mechanical characteristics and permeability porosity, and six test blocks were prepared for each mix proportion. A part of these test blocks is depicted in Figure 5. Under identical conditions, the performance of these blocks will be evaluated after a curing period of 28 days; during this period, geotextiles were laid on the concrete test block to reduce the rate of water evaporation, and water was sprinkled on the geotextile daily, as illustrated in Figure 6.

### 2.4. Testing Method

#### 2.4.1. Porosity and Density

The porosity and density were tested for all the blocks. The porosity was tested according to the relevant methods in the guideline CJJ/T135-2009 [30] as follows. Firstly, taking out the blocks to be tested after curing for 28 days, wiping the surface moisture, testing and recording the section size of the blocks, drying them in the oven for 24 h, and keeping the oven temperature at 60 °C. Secondly, cooling the test blocks to room temperature in a dryer, and weighing and recording the mass *m*_1_ of them. Thirdly, immersing the blocks after being weighed for 24 h. Fourth, weighing the mass *m*_2_ of the test blocks in water, where the water level should be about 20 cm higher than the test blocks, and calculating the porosity according to the following formula:

(1) $$p=1-\frac{m_{2}-m_{1}}{\rho_{w}V_{0}}$$

where *p* denotes the porosity; *ρ_w_* denotes the density of water; and *V*_0_ denotes the volume of the block.

The density of concrete block *ρ* could be tested by the following formula:

(2) $$\rho =\frac{m_{1}}{V_{0}}$$

#### 2.4.2. Compressive Strength

**Figure 7:**
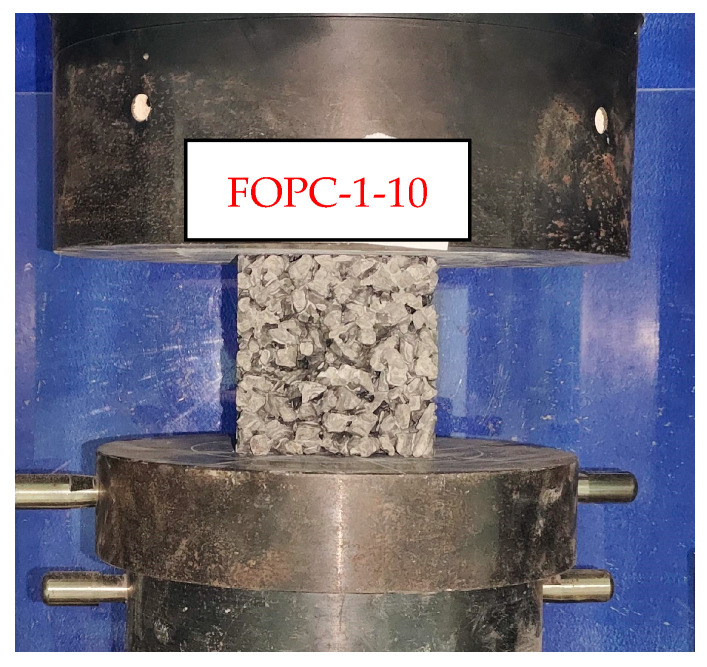
Test of the compressive strength of FOPC.

The compressive strengths of the FOPC were determined using a pressure testing machine, as illustrated in Figure 7, employing a loading rate of 0.4 mm/s. To ascertain the ultimate load, the blocks were completely crushed, thereby enabling the calculation of the corresponding compressive strength. After obtaining the peak force $F_{peak}$ of concrete, its compressive strength can be obtained by the following equation,

(3) $$f_{c}=0.95\frac{F_{peak}}{A}$$

#### 2.4.3. Permeability Coefficient

**Figure 8:**
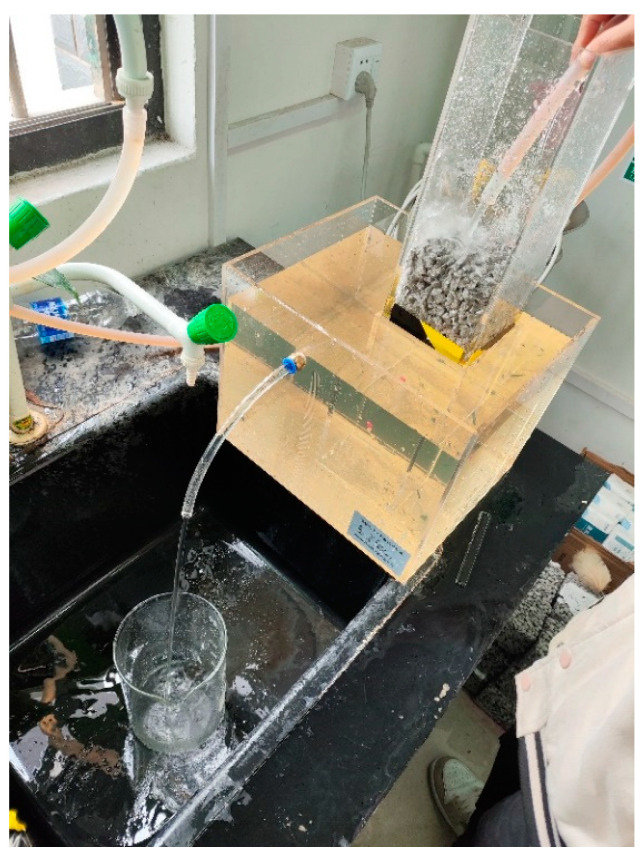
Experimental set-up for measuring the permeability coefficients for FOPC.

The size of the block is 100 mm × 100 mm × 100 mm. Before the start of the test, in order to prevent water from overflowing from around the FOPC, the waterproof mud adhesive was used to smooth the edges of the FOPC, ensuring that water can only flow from the top and bottom surfaces. The permeability coefficient of the pervious concrete shall be tested and measured in accordance with the requirements of guideline CJJ/T135-2009 [30]. The experimental set-up for measuring permeability coefficients is shown in Figure 8, and the process of measuring the permeability coefficient is as follows: first, after 28 days of curing of the test block, the lengths of the upper surface of the pervious concrete test blocks are measured with a ruler, and the upper surface area *A* is calculated; then, the test block is immersed in water until it reaches saturation and is put into the testing set-up. Water is injected into the testing set-up at a constant speed until the water can overflow from the sleeve overflow port and overflow tank overflow port. After the water flow stabilizes, timing is started and the time taken by the measuring cylinder to weigh 1 L of water is recorded, taking the average of three measurements as the experimental result. The formula used to calculate the permeability coefficient, *k*, is expressed as follows [5,31,32]:

(4) $$k=\frac{QL}{AHt}$$

In this equation, *k* signifies the quantity of water that seeps through in time *t*; *Q* denotes the water output within *t* time; *L* stands for the specimen’s height; *A* represents the cross-sectional area; *H* denotes the water level difference; and *t* refers to the time elapsed.

## 3. Results and Discussion

### 3.1. Porosity and Density

**Figure 9:**
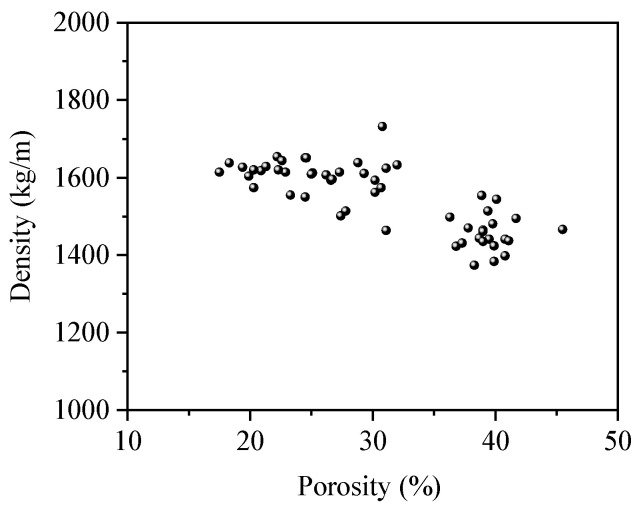
The relationship between density and porosity.

The interrelation of density and porosity is depicted in Figure 9. The porosity of these blocks ranged from 15% to 45%, aligning with the findings of Mitrosz et al. [12]. The density varied between 1400 and 1700 kg/m^3^. Generally, an inverse relationship was observed between the density and porosity of the test blocks, albeit with considerable dispersion.

### 3.2. Strength Test of FOPM

**Figure 10:**
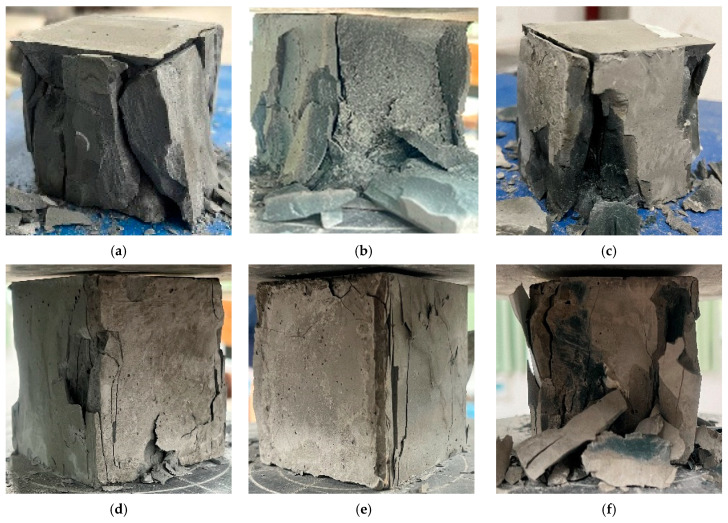
Failure mode of the FOPM test blocks: (**a**) FOPM-0; (**b**) FOPM-10; (**c**) FOPM-20; (**d**) FOPM-30; (**e**) FOPM-40; (**f**) FOPM-50.

For the preparation of FOPC, OPC, FNS, reinforcing agent, and water were combined to form the FOPM. The compressive strength of this FOPM was determined using pressure testing machines. Figure 10 illustrates the failure modes of FOPM blocks with varying FNS substitution rates. It is observed that the internal and external colors and shapes of FOPM with different FNS substitution rates were basically consistent; the failure modes were basically consistent and similar to that of ordinary cementitious materials.

**Figure 11:**
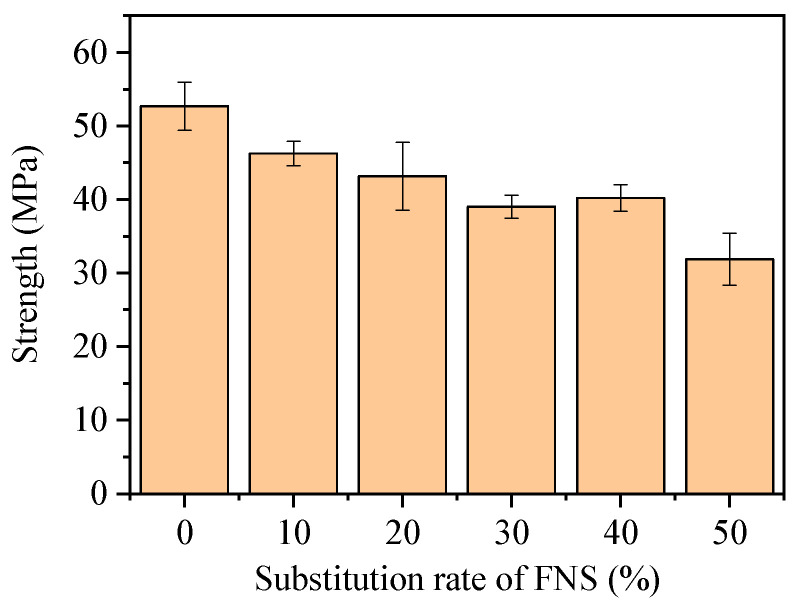
Compression strength of the FOPM.

**Figure 12:**
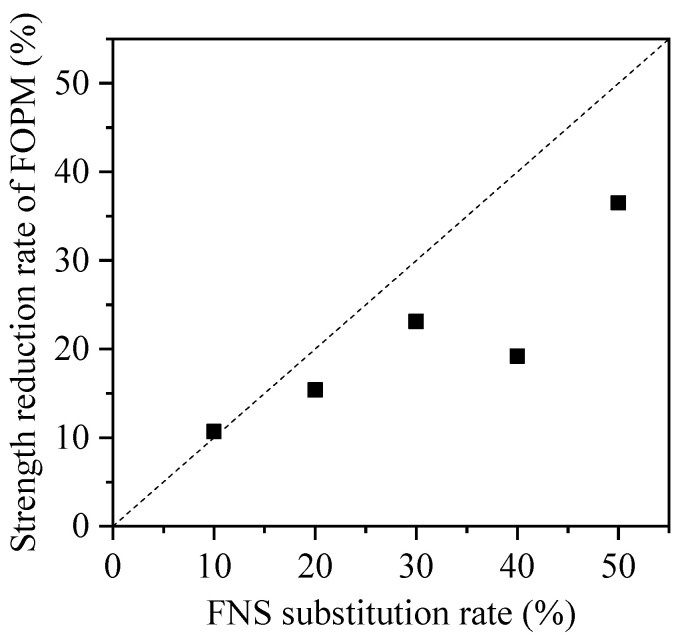
Comparison between the FNS substitution rate and the strength reduction rate of FOPM.

Figure 11 displays the strengths and associated dispersions of FOPM specimens with varying FNS substitution rates. Generally, there is a decrease in the strength of these specimens correlating with an increase in FNS substitution rate. Specifically, when the FNS substitution rate is at 10%, 20%, 30%, 40%, and 50%, the strength reduction in the FOPM is observed to be 10.7%, 15.4%, 23.1%, 19.2%, and 36.5%, respectively, as shown in Figure 12; when the FNS substitution rate is relatively small, FNS does not make a significant contribution to strength. However, as the FNS substitution rate increases, the strength reduction rate of the FOPM is inconsistent with the FNS substitution rate, and the strength reduction rate is smaller than the FNS substitution rate. The phenomenon of non-proportional reduction indicates that FNS plays a strength contribution role in cementitious materials. This is mainly due to the alkaline substances of cement stimulating the activity of FNS during the mixing process with cement and water, thereby forming a cementitious effect.

### 3.3. Strength of FOPC

**Figure 13:**
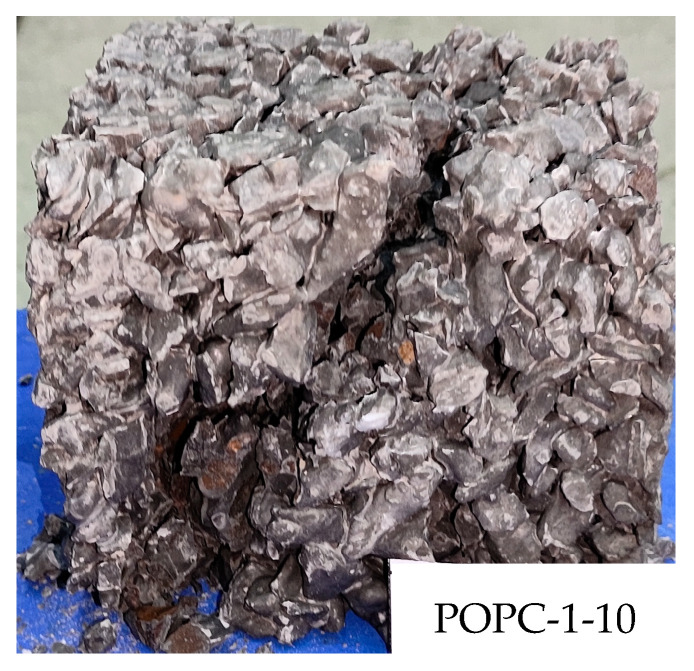
Failure mode of FOPC.

Due to the lack of fine aggregates, from a macro perspective, the strength of POPC depends on the strength of FOPM, aggregate strength, and the interface strength between FOPM and aggregate. The strength of aggregates is usually high, and the porosity of permeable concrete is large. The interaction between aggregates is small, so the interface strength between FOPM and aggregates plays a decisive role in the strength of FOPM. The failure mode of the test block during the test also proves this point, and Figure 13 depicts the failure mode of the test blocks. It can be seen that the crushed test block has multiple cracks and one main crack, with some aggregates falling off next to the main crack due to FOPM damage.

**Figure 14:**
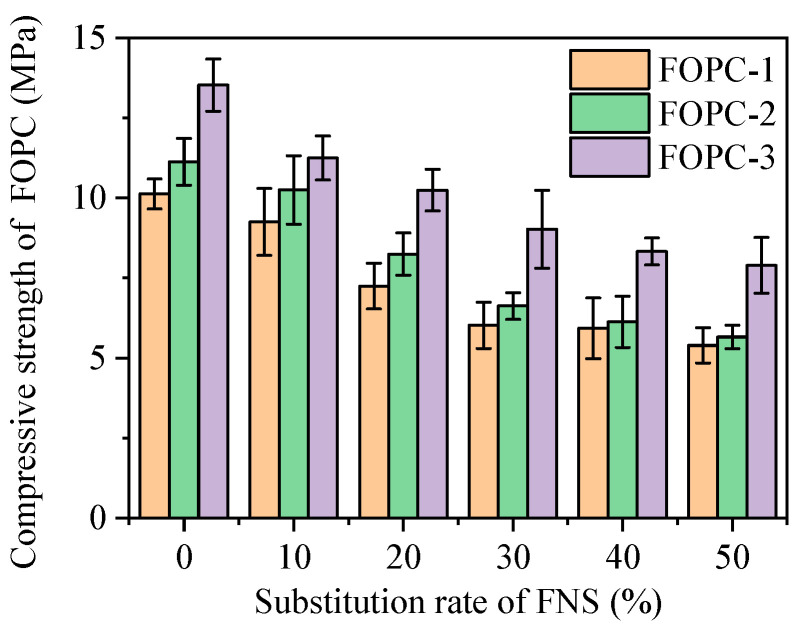
Compressive strength of FOPC.

Displayed in Figure 14 are the findings regarding the compressive strength of FOPC with different mixing proportions. In scenarios devoid of FNS, the strength of the FOPC was observed to lie between 10 and 14 MPa. With the incorporation of varying levels of FNS, the strength varied, showing a range from 6 to 14 MPa. This also means that in the same ratio of paste material to aggregate, the addition of FNS will reduce the strength of FOPC. According to the description in Section 3.2, an increase in the proportion of FNS will reduce the strength of the FOPM.

Comparing the changes in FOPC strength with the ratio of FOPM to aggregate, it can be found that under the same FNS substitution rate, the FOPC strength increases with the increase in the ratio of FOPM to aggregate. When the ratio is increased from 0.1875 (FOPC-1 group) to 0.2000 (FOPC-2 group), that is, an increase of 6.7%, the FOPC strength increases by 10–13%, and when the ratio is increased from 0.1875 (FOPC-1 group) to 0.2188 (FOPC-3 group), that is, an increase of 16.5%, the FOPC strength increases by 30–50%. These results highlight the pivotal role of cementitious material content in determining the strength of FOPC, indicating that the higher strength of the FOPMs lead to stronger FOPC.

The inclusion of varying FNS levels across distinct mix ratios revealed a consistent reduction in the compressive strength of FOPC with the increment of FNS substitution rate. For instance, in the FOPC-1 group, increasing FNS substitution from 10 to 50% sequentially diminished FOPC strength by 8.7%, 28.5%, 40.5%, 41.5%, and 46.7%. For FOPC-2 group, escalating the FNS substitution rate from 10 to 50% led to reductions in strength by 7.9%, 25.9%, 40.5%, 44.9%, and 49.2%, correspondingly. With the FOPC-3 group, boosting FNS substitution rates from 10 to 50% incurred strength decreases of 16.8%, 24.3%, 33.3%, 38.4%, and 41.6%, respectively. These trends highlight the pivotal role of the strength of FOPM, which declines with an increased FNS substitution rate, influencing FOPC’s compressive capabilities. Additionally, the mix proportions significantly impact FOPC strength; enriching the mixture with more cementitious materials tends to elevate FOPC strength. This enhancement is linked to the unique composition of FOPC, which exclusively comprises coarse aggregates and cementitious materials, where a higher concentration of cementitious components improves coverage around coarse aggregates, thus, elevating the FOPC’s compressive strength.

**Figure 15:**
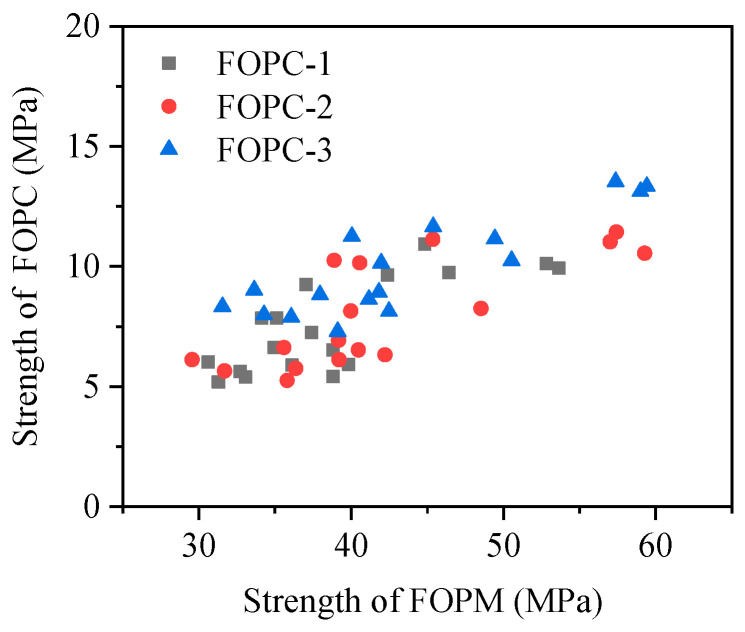
Compressive strength of FOPC vs. FOPM.

The compressive failure of FOPC is mainly due to the failure of the interface between FOPM and aggregate. The interface strength between FOPM and aggregate plays a decisive role in the compressive strength of FOPC, while the strength of cementitious materials has a significant impact on the interface strength. Figure 15 depicts the association between FOPM strength and FOPC compressive strength. The trend indicates a general increase in FOPC strength with the enhancement of FOPM strength, following a linear pattern, albeit with significant scatter among the data points.

**Figure 16:**
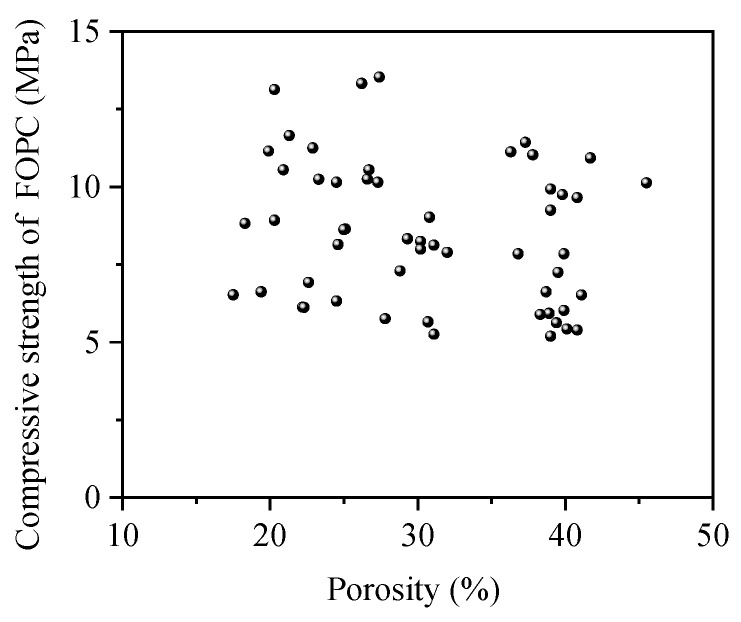
Compressive strength of FOPC vs. porosity.

Figure 16 illustrates the link between the porosity of FOPC and its strength. It reveals that there is no substantial connection between FOPC’s strength and its porosity, with a noticeable spread in the data. This observation is mainly due to the FOPC strength being dependent on factors beyond porosity, particularly the strength of the FOPMs used.

### 3.4. Permeability Performance

**Figure 17:**
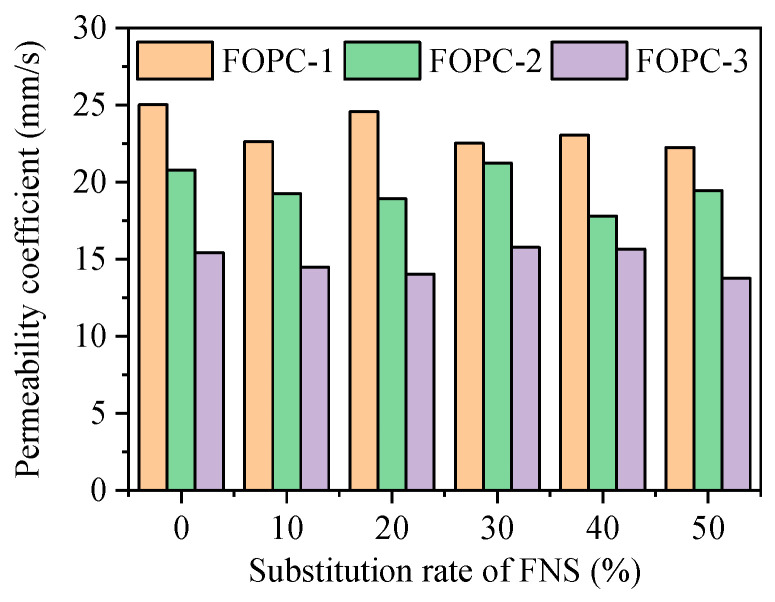
Permeability coefficient of FOPC.

Displayed in Figure 17 are the findings regarding the permeability coefficient of FOPC with different mixing proportions. The permeability coefficient of the FOPC was observed to lie between 13 and 26 mm/s. It can be seen that the substitution rate of FNS has a relatively small impact on the permeability coefficient and there is no obvious pattern. The concrete mix proportion, which is the ratio of aggregates to cementitious materials, has a significant impact on the permeability coefficient.

The permeability coefficient of the FOPC-1 group is the highest, with a ratio of 0.1875 between FOPM and aggregate; the permeability coefficient of the FOPC-2 group is second, with a ratio of 0.2000; and the permeability coefficient of the FOPC-3 group is the smallest, with a ratio of 0.2188. This indicates that the ratio of FOPM to aggregates has a significant impact on the permeability coefficient, as the ratio of FOPM to aggregates directly affects the porosity, which in turn affects the permeability coefficient.

**Figure 18:**
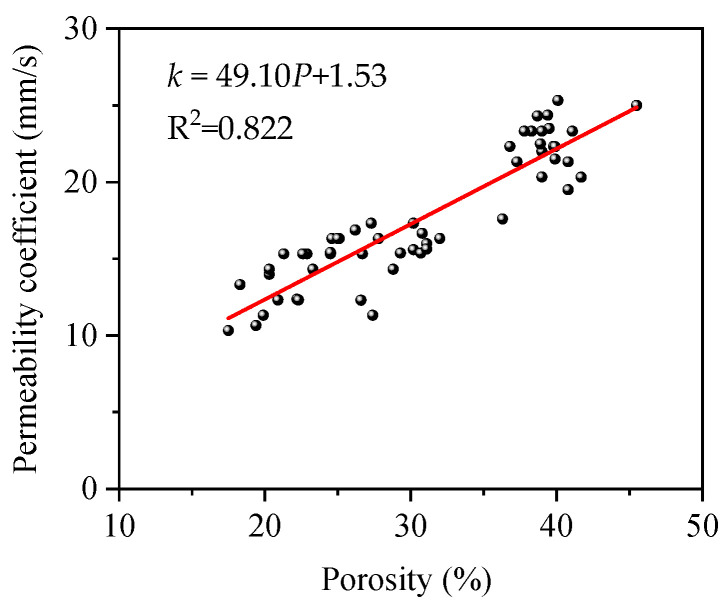
Permeability coefficients vs. the porosity of FOPC.

FOPC’s permeability characteristics largely hinge on porosity, with Figure 18 illustrating the correlation between porosity and the permeability coefficient. This coefficient usually spans from 10 to 25 mm/s, consistent with observations in references [33,34]. As porosity escalates, the permeability coefficient also tends to rise, generally following a linear trajectory but exhibiting scatter. This scatter is attributed to the heterogenous distribution of aggregates and FOPMs, affecting the water’s passage through the FOPC. By linear fitting, the relationship between the permeability coefficient and porosity can be obtained as follows:

(5) k=49.10P+1.53

where *P* denotes the porosity of FOPC; *k* is the permeability coefficient; and the unit is mm/s. The fitting result can be seen in Figure 18, with an R^2^ of 0.822. Although there is a discrete type, the permeability coefficient can be roughly calculated based on the porosity.

## 4. Conclusions

Exploring the feasibility of FOPC included assessing FNS’s basic attributes, the potency of FOPMs, along with the FOPC’s mechanical, physical, and permeability coefficients. The key conclusions are as follows:

(1) The chemical composition and particle dimensions of FNS align with those of mineral admixtures like Metakaolin, mainly containing CaO, SiO_2_, Al_2_O_3_, MgO and other substances, and after grinding into powder, the particle size distribution of FNS is within the range of 0.5 to 100 μm.
(2) As the replacement rate of FNS increases, the strength of FOPM does not decrease proportionally, which also indicates that FNS plays a strength contribution role in cementitious materials.
(3) The presence of FOPM plays a crucial role in determining the strength of FOPC, and when the strength of FOPM and the ratio of cementitious to aggregate increases, both can improve the strength of FOPC. When the ratio of cementitious to aggregate increased by 6.7% and 16.5%, the strength of FOPC increased by 10–13% and 30–50%, respectively.
(4) The ratio of cementitious to aggregates markedly impacts the permeability coefficient. This is because the ratio of FOPM to aggregate has a significant impact on porosity, which indirectly affects the permeability coefficient, and the FOPC’s permeability coefficient increases with porosity, predominantly exhibiting a linear progression, albeit with some inconsistencies. In design, porosity can be controlled to control the permeability coefficient.
(5) FOPC has excellent performance in terms of permeability, and its strength is lower than that of ordinary pervious concrete. Therefore, FOPC can be used on some road surfaces with lower strength requirements. In addition, due to its high porosity, FOPC can also be attempted for use in vegetation concrete. Due to the certain activity of FNS, future work will also attempt to stimulate its activity through alkali to obtain better performance cementitious materials.

## Figures and Tables

**Table 1:** Components of FNS.

Component	CaO	SiO_2_	Al_2_O_3_	MgO	SO_3_	MnO	Others
%	35.58	30.91	16.71	10.27	2.25	1.12	3.16

**Table 2:** Mix proportions of FOPC.

Group	No.	Coarse Aggregate (kg)	Cementitious Material (kg)		Reinforcing Agent (kg)	Water (kg)
			OPC	FNS		
FOPC-1	FOPC-1-0	1600	300	0	10	136
	FOPC-1-10	1600	270	30	10	136
	FOPC-1-20	1600	240	60	10	136
	FOPC-1-30	1600	210	90	10	136
	FOPC-1-40	1600	180	120	10	136
	FOPC-1-50	1600	150	150	10	136
FOPC-2	FOPC-2-0	1600	320	0	12	144
	FOPC-2-10	1600	288	32	12	144
	FOPC-2-20	1600	256	64	12	144
	FOPC-2-30	1600	224	96	12	144
	FOPC-2-40	1600	192	128	12	144
	FOPC-2-50	1600	160	160	12	144
FOPC-3	FOPC-3-0	1600	350	0	15	154
	FOPC-3-10	1600	315	35	15	154
	FOPC-3-20	1600	280	70	15	154
	FOPC-3-30	1600	245	105	15	154
	FOPC-3-40	1600	210	140	15	154
	FOPC-3-50	1600	175	175	15	154

**Table 3:** Mix proportions of FOPM.

No.	Cementitious Material (kg)		Water (kg)
	OPC	FNS	
FOPM-0	300	0	136
FOPM-10	270	30	136
FOPM-20	240	60	136
FOPM-30	210	90	136
FOPM-40	180	120	136
FOPM-50	150	150	136

Note: The number following FOPM denotes the substitution rate (%) of FNS.

## Data Availability

Data are contained within the article.
